# Six degrees head-down tilt bed rest caused low-grade hemolysis: a prospective randomized clinical trial

**DOI:** 10.1038/s41526-021-00132-0

**Published:** 2021-02-15

**Authors:** Kathryn Culliton, Hakim Louati, Odette Laneuville, Tim Ramsay, Guy Trudel

**Affiliations:** 1 Department of Medicine, Division of Physical Medicine and Rehabilitation, Ottawa Hospital Research Institute, Ottawa, ON Canada; 2 Department of Biology, Faculty of Science, University of Ottawa, Ottawa, ON Canada; 3 School of Epidemiology and Public Health, University of Ottawa, Ottawa, ON Canada; 4 Department of Biochemistry, Microbiology, and Immunology, University of Ottawa, Ottawa, ON Canada

**Keywords:** Epidemiology, Randomized controlled trials

## Abstract

This study aimed to measure hemolysis before, during and after 60 days of the ground-based spaceflight analog bed rest and the effect of a nutritional intervention through a prospective randomized clinical trial. Twenty male participants were hospitalized for 88 days comprised of 14 days of ambulatory baseline, 60 days of 6° head-down tilt bed rest and 14 days of reambulation. Ten participants each received a control diet or daily polyphenol associated with omega-3, vitamin E, and selenium supplements. The primary outcome was endogenous carbon monoxide (CO) elimination measured by gas chromatography. Hemolysis was also measured with serial bilirubin, iron, transferrin saturation blood levels and serial 3-day stool collections were used to measure urobilinoid excretion using photometry. Total hemoglobin mass (tHb) was measured using CO-rebreathing. CO elimination increased after 5, 11, 30, and 57 days of bed rest: +289 ppb (95% CI 101–477 ppb; *p* = 0.004), +253 ppb (78–427 ppb; *p* = 0.007), +193 ppb (89–298 ppb; *p* = 0.001) and +858 ppb (670–1046 ppb; *p* < 0.000), respectively, compared to baseline. Bilirubin increased after 20 and 49 days of bed rest +0.8 mg/l (*p* = 0.013) and +1.1 mg/l (*p* = 0.012), respectively; and iron increased after 20 days of bed rest +10.5 µg/dl (*p* = 0.032). The nutritional intervention did not change CO elimination. THb was lower after 60 days of bed rest −0.9 g/kg (*p* = 0.001). Bed rest enhanced hemolysis as measured through all three by-products of heme oxygenase. Ongoing enhanced hemolysis over 60 days contributed to a 10% decrease in tHb mass. Modulation of red blood cell control towards increased hemolysis may be an important mechanism causing anemia in astronauts.

## Introduction

Space anemia after exposure to microgravity was first observed in six astronauts from Gemini missions^1^. Later studies correcting for environmental oxygen concentrations failed to resolve the anemia in 4 returning astronauts from the Spacelab 1 mission^2^. Further experiments identified a 10–15% decrease in erythrocyte mass measured in three astronauts after 9 days^3^ and in six astronauts after 9 and 14 days in space^4^. More recently, longer-duration space missions followed erythropoietic adaptation to space after the initial 10 days and reported no anemia onboard the ISS throughout 6-month missions questioning whether space anemia was real or had been resolved with modern space travel^5^. However, epidemiological data from over 5 decades of American and Canadian presence in space confirmed and characterized space anemia^6^.

The search for mechanisms to explain space anemia has generated multiple hypotheses and experiments over the past decades such as: ineffective erythrocyte production and/or egress from the bone marrow^3,7,8^, low erythropoietin levels or sensitivity^3,4^, and production of abnormally-shaped erythrocytes^9^. In situations of hemoconcentration the excess erythrocytes can be sequestered in the spleen^7^, remain in circulation until time-determined senescence^3^, or their lifespan can be shortened preferentially affecting young erythrocytes^4^ or equally affecting erythrocytes of all ages^10^.

Hemolysis regulates erythropoietic homeostasis^11–13^. Alfrey proposed that upregulated hemolysis explained the decrease in total hemoglobin mass (tHb) using data obtained by labeling erythrocytes^3,4^. However, this evidence for hemolysis was indirect^14^. Volunteers exposed to bed rest with 6° head-down tilt (HDT) also displayed lower tHb upon reambulation^15–17^. Clinically, an abundance of data demonstrates an association between bed rest and anemia^18–35^. However, there has been debate over whether this might be caused by a reduction in erythropoiesis, an increase in hemolysis, or both. Our trial sought to answer that question. In parallel, the effectiveness of a nutritional intervention of food plants, vitamin E and omega-3 at preventing deconditioning induced by bed rest was investigated. Its effect on hemolysis is unknown and was assessed^36–38^.

Hemolysis can be quantified directly by measuring degradation products of hemoglobin. Senescent red blood cells (RBC) release hemoglobin molecules. Each of the four heme rings is enzymatically degraded by heme oxygenases (HO)^11^ producing equimolar amounts of CO, biliverdin and ferrous ion^12,13^. Hemoglobin heme is the largest source of endogenous CO (85%), which allows attributing changes in CO elimination at the lungs to changes in the steady-state rate of hemolysis^39,40^. CO elimination is the difference between alveolar and ambient CO concentrations ([CO]) at a stable ventilation rate. Each molecule of biliverdin from heme degradation is conjugated in the liver to bilirubin, excreted in the intestine, converted to urobilinoids and eliminated in the feces^41^. Finally, serum iron levels and iron-related proteins can be measured in the serum.

Our objectives were to assess: (1) the effect of exposure to 60 days of bed rest HDT, and (2) the effect of a nutritional intervention on hemolysis in 20 volunteers using carbon monoxide (CO) elimination as the primary outcome; (3) To assess the effect of hemolysis on tHb mass. Our hypotheses were that: (1) exposure to bed rest HDT will increase hemolysis while (2) a nutritional intervention will have no effect; and (3) prolonged durations of increased hemolysis will contribute to anemia.

## Results

**Fig. 1:**
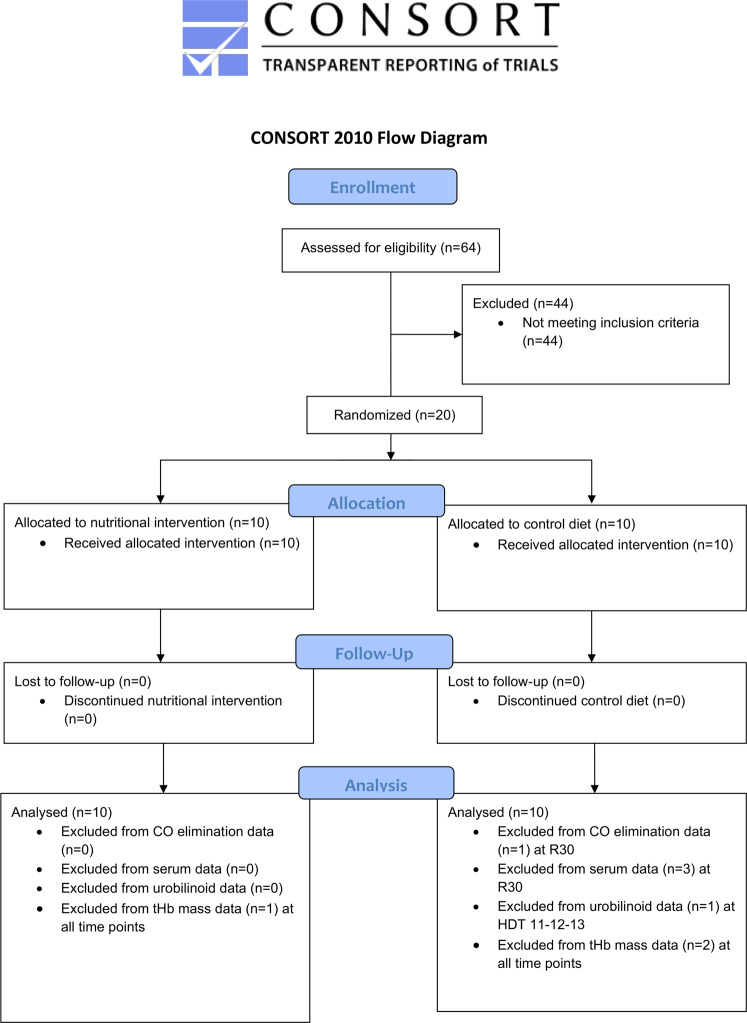
CONSORT flow diagram for transparent reporting of trials. CO data from 1 participant at R30 was excluded due to admitted environmental factors (cigarette smoking). Blood was not drawn on three participants at reambulation Day 30. Stool samples were not collected for one participant at HDT11,12,13. Total Hb mass determination was excluded for three participants due to technical difficulties.

All participants (mean age 34.2 [SD, 7.8] years) completed the study (Fig. 1). There was no effect of the nutritional intervention on hemolysis (Supplementary Table 1 and Supplementary Fig. 1). Therefore, participants’ data were combined (*n* = 20) to analyze the effect of exposure to bed rest on hemolysis.

### Hemolysis markers

**Fig. 2:**
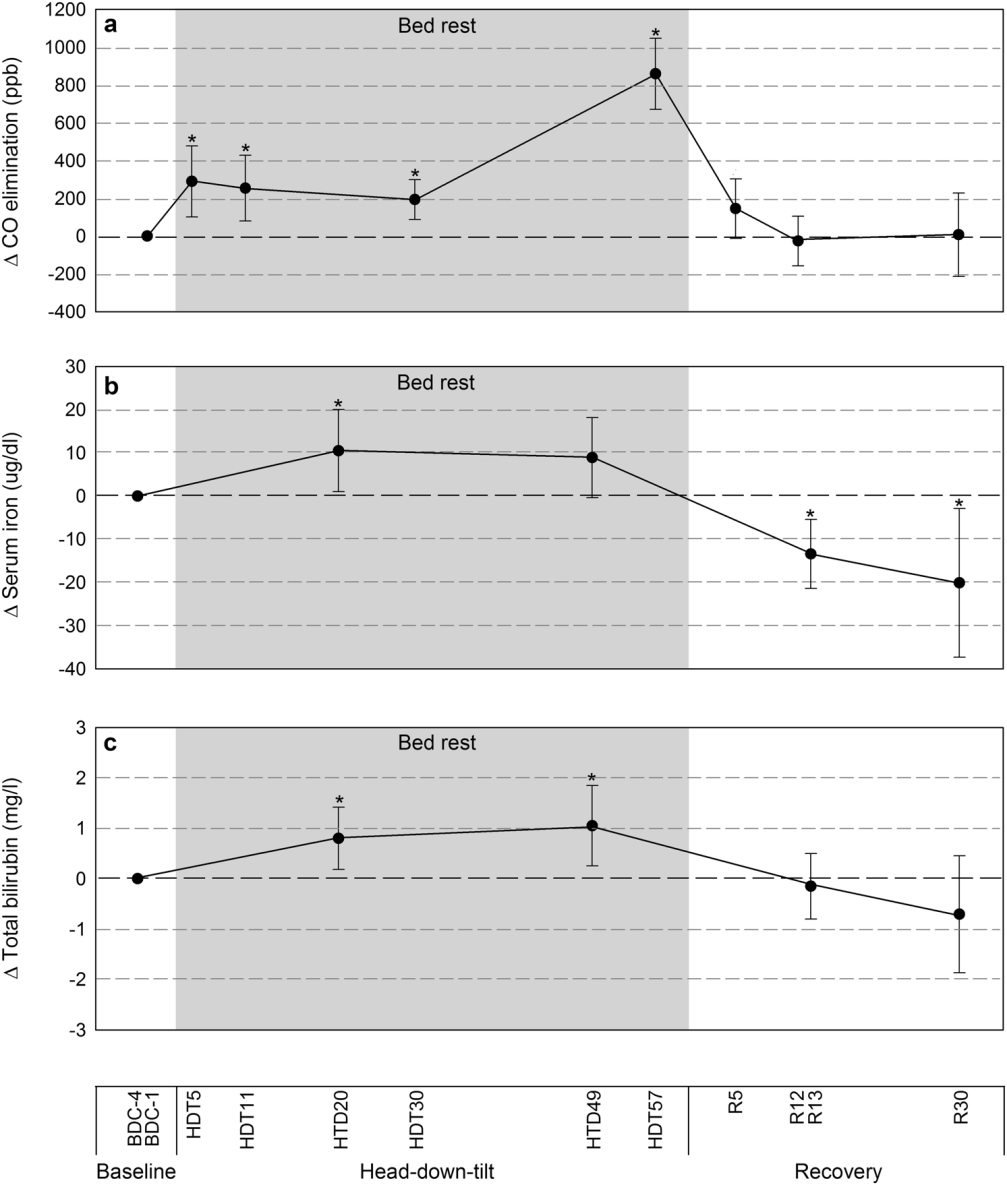
Measures of the three by-products of heme degradation by the hemoglobin oxygenase enzymes in 20 male participants before, during and after 60 days of bed rest HDT. **a** Endogenous CO elimination was elevated at all time points during bed rest. **b** Iron levels were elevated after 20 days of bed rest and decreased below baseline after bed rest. **c** Bilirubin was elevated at all time points during bed rest. These findings support low-grade hemolysis with enforcing and maintaining the bed rest position. Data expressed as changes from baseline. Shaded area represents the bed rest HDT phase. BDC baseline data collection, HDT head-down tilt, R recovery or reambulation. Error bars: 95% confidence interval. *p < 0.05 compared to baseline.

Baseline CO elimination was 1795ppb (95% CI: 1644–1947ppb; Table 2). CO elimination was higher after 5, 11, 30, and 57 days of bed rest compared to baseline: +289 ppb (101–477 ppb), +253 ppb (78–427 ppb), +193 ppb (89–298 ppb) and +858 ppb (670–1046 ppb), respectively; (Fig. 2a). The average increase in CO elimination during bed rest was 23%. CO elimination measured at reambulation days 5, 12, and 30 were comparable to baseline. Results from the two campaigns were comparable (Supplementary Fig. 2).

Iron was higher after 20 days of bed rest: +10.5 µg/dl (1.0–19.9 µg/dl) compared to baseline (Fig. 2b). The average increase in serum iron during bed rest was 14%. Iron was lower than baseline at reambulation days 13 and 30 (Fig. 2b and Table 1).

**Table 1:** Quantification of hemolysis using measures of heme degradation products.

Assay	Bed rest study timepoint													
	BDC-13	BDC-4	BDC-1	HDT5	HDT11	HDT20	HDT30	HDT49	HDT56	HDT57	R5	R12	R13	R30
CO ppb			1795 (1644–1947)	**2085 (1900–2270)**	**2048 (1808–2288)**		**1988 (1790–2187)**			**2653 (2409–2898)**	1940 (1707–2174)	1768 (1591–1945)		1799 (1551–2048)*
Bilirubin mg/l		6.7 (5.4–8.0)				**7.5 (6.0–9.0)**		**7.8 (6.2–9.3)**					6.6 (5.4–7.7)	5.2 (4.1–6.3)**
Urobilinoid mg/24 h	326 (260–393)				410 (311–509) *		360 (286–433)		376 (275–476)		335 (243–426)			
Iron µg/dl		81.4 (72.3–90.5)				**91.8 (79.7–104.0)**		90.2 (80.8–99.7)					**67.9 (57.9–78.0)**	60.5 (47.5–73.5)**
Transferrin saturation %		29.0 (24.7–33.2)				31.4 (26.5–36.4)		**33.1 (28.7–37.6)**					**25.2 (21.2– 29.1)**	20.1 (15.7–24.4)**

Data (95% CI) for hemolysis markers before, during, and after bed rest. Underlined values show the bed rest phase. Bold data were statistically significant from baseline (*p* < 0.05). *n = 19, **n = 17.

Total bilirubin was higher after 20 and 49 days of bed rest compared to baseline, +0.8 mg/l (0.2–1.4 mg/l) and +1.1 mg/l (0.3–1.8 mg/l), respectively (Fig. 2c). The average increase in total bilirubin during bed rest was 19%.

Urobilinoids elimination was higher after 11-12-13, 30-31-32, and 56-57-58 days of bed rest: +75.9 mg/day (−15.4–167.2 mg/day), +33.3 mg/day (−31.0–97.5 mg/day) and +49.2 mg/day (−29.6–128.0 mg/day), respectively (Table 1) but the confidence intervals included zero. The average increase in urobilinoids elimination during bed rest was 20%.

The transferrin saturation was higher after 49 days of bed rest: +4.1% (0.3–8.0%; Table 1). The average increase in transferrin saturation during bed rest was +15%. Transferrin saturation was lower at reambulation days 13 and 30. Haptoglobin was unchanged throughout bed rest (Table 2).

**Table 2:** Erythropoietic markers. Data (95% CI) for erythropoietic markers before, during, and after bed rest.

Measure	Bed rest study timepoint							
	BDC-4	HDT20	HDT49	HDT60	R1	R7	R13	R30
RBC 10^6^/mm^3^	4.529 (4.393–4.665)	**5.011 (4.887–5.134)**	**5.007 (4.834–5.179)**	**4.803 (4.627–4.979)**	4.469 (4.322–4.615)		**4.348 (4.180–4.516)**	4.358 (4.168–4.547)**
Reticulocyte 10^3^/µl	47.4 (39.1–55.6)	50.7 (41.2–60.3)	45.9 (38.9–52.9)				**63.9 (55.9 – 71.9)**	56.6 (48.6–64.6)**
tHb g/kg	10.9 (10.4–11.5)**			10.0 (9.5–10.5)**		9.6 (9.1–10.1)**		
tHb g	818.2 (764.4–872.0)**			735.0 (673.9–796.0)**		723.5 (674.6–772.5)**		
Hb g/dl	13.7 (13.4–14.0)	**15.1 (14.8–15.4)**	**15.0 (14.6–15.5)**	**14.4 (13.9–14.8)**	13.4 (13.0–13.8)		**13.1 (12.7–13.5)**	**13.2 (12.7–13.6)**
Participants Hb < 13.0 g/dl	2/20	**0/20**	**0//20**	**0/20**	5/20		**9/20**	**7/17**
EPO mUI/ml	12.2 (10.2–14.2)	**8.9 (7.7–10.1)**	**10.1 (8.7–11.6)**				**16.3 (12.8 – 19.9)**	17.8 (13.6–22.0)**
Haptoglobin g/l	1.03 (0.89–1.16)	0.99 (0.87–1.12)	1.04 (0.89–1.19)				1.07 (0.94–1.21)	1.10 (0.93–1.27)**

Underlined values show the bed rest phase. Bold data were statistically significant from baseline (*p* < 0.05). **n = 17.

### Erythropoietic markers

**Fig. 3:**
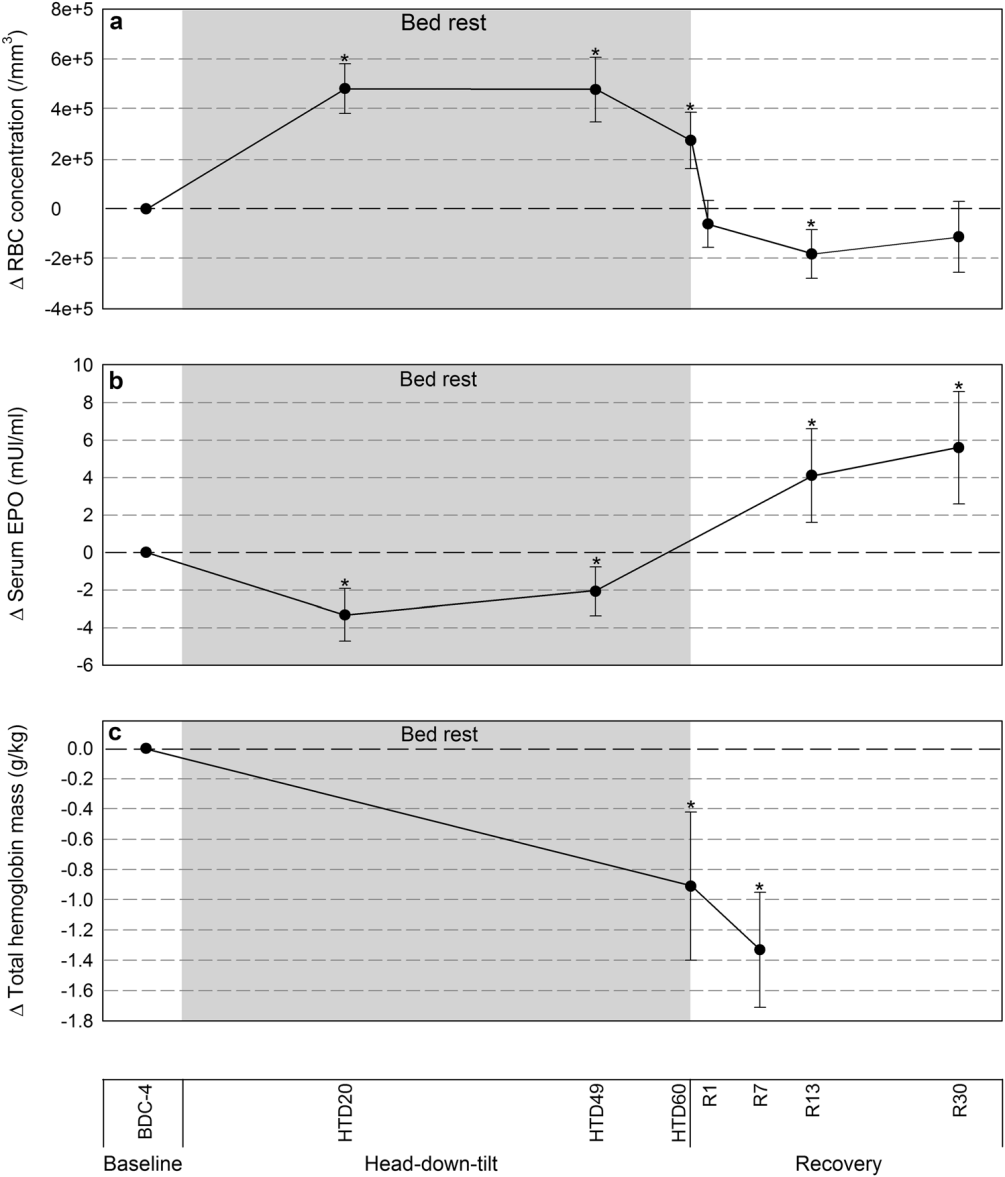
Erythropoietic changes in 20 male participants before, during and after 60 days of bed rest HDT. **a** Participants remained hemoconcentrated at all time points during bed rest. Reambulation uncovered lower RBC concentration. **b** EPO was decreased at all time points during bed rest. EPO increased at reambulation. **c** Total hemoglobin mass was decreased after 60 days of bed rest and at recovery Day 7. Sixty days of bed rest caused a 77.2 g loss in tHb mass attributed to hemolysis and blood draws. Data expressed as changes from baseline. Shaded area represents the bed rest HDT phase. BDC: Baseline Data Collection; HDT: Head-Down Tilt; R: Recovery or Reambulation. Error bars: 95% Confidence Interval. *p < 0.05 compared to baseline.

Red blood cell concentration was higher after 20, 49, and 60 days of bed rest: +48.2 10^4^/mm^3^ (3.8–5.8 10^4^/mm^3^); +47.8 10^4^/mm^3^ (3.5–6.1 10^4^/mm^3^), and +27.4 10^4^/mm^3^ (1.6–3.9 10^4^/mm^3^), respectively, compared to baseline (Fig. 3a). The average increase in red blood cell concentration during bed rest was 9%. Red blood cell concentration was lower at reambulation day 13 (Fig. 3a and Table 2). Mean hemoglobin concentrations were −0.55 g/dl (−0.9 to −0.2 g/dl) and −0.46 g/dl (−0.9 to −0.1 g/dl) at reambulation days 13 and 30, respectively (Table 2).

EPO was lower after 20 and 49 days of bed rest: −3.3 mUI/ml (−4.7 to −2.0 mUI/ml), and −2.1 mUI/ml (−3.4 to −0.7 mUI/ml); respectively, compared to baseline (Fig. 3b). The average decrease in EPO during bed rest was 18%. EPO was higher than baseline at reambulation days 13 and 30.

Total hemoglobin mass was significantly lower after 60 days of bed rest and at reambulation day 7: −0.9 g/kg (−1.4 to −0.4 g/kg) and −1.3 g/kg (−1.7 to −1.0 g/kg), respectively, compared to baseline (Fig. 3c). The average decrease in tHb mass between baseline and 60 days of bed rest was 10% (Table 2).

Reticulocyte concentrations were unchanged during bed rest (Table 2) but were higher than baseline at reambulation days 13 and 30: +16.6 10^3^/µl (0.9–2.4 10^3^/µl) and +10.8 10^3^/µl (0.3–1.9 10^3^/µl), respectively.

## Discussion

This prospective randomized clinical trial provided the first direct confirmation of enhanced hemolysis using the bed rest space analog model. In healthy men, CO elimination increased on average 23%. The other heme degradation products were also elevated throughout bed rest (bilirubin + 19%, urobilinoids +20%, iron +14% and transferrin saturation +15%). The difference in percent increase between the 3 by-products of heme oxygenase may be explained by their individual metabolic pathways. Bilirubin clearance accrues when production increases,^42^ in addition, the buffering of iron by various proteins^43^ may also decrease their levels. CO is not further degraded and constitutes a reliable marker of hemolysis^44^. Participants’ enhanced hemolysis was synchronous with the bed rest position. All hemolysis markers increased from the first measures, remained elevated throughout the 60 days of bed rest, and returned to or below baseline levels at reambulation. Taken together, the increased levels of the three heme by-products in synchrony with bed rest confirmed our first hypothesis that exposure to bed rest HDT (head-down-tilt) increases hemolysis. The nutritional intervention designed to prevent deconditioning showed no effect on hemolysis, which confirmed the second hypothesis.

Sustained increased hemolysis can contribute to anemia. The participants in the current study experienced a mean 10% decrease in tHb mass at day 60 of bed rest. In fact, the hemoglobin concentration of 9/20 participant reached clinical anemia levels^45^ at day 13 of reambulation (Table 2) confirming our third hypothesis. Increased hemolysis during bed rest in the HDT position may be related to low EPO, bone marrow unloading and circadian cycle.

Increased hemolysis was associated with an 18% decrease in EPO levels throughout bed rest. Low EPO levels have also been consistently reported with space missions^46^. They were interpreted as a response to hemoconcentration that triggered peripheral hemolysis of young RBC to rapidly return RBC concentration to normal levels^4,10,46–48^. In this trial, participants remained hemoconcentrated (+9%), however, the unchanged reticulocyte concentration argued against their preferential hemolysis. Also, low EPO, hemoconcentration, and increased hemolysis persisted for 60 days and were not a rapid adaptation to the initial days of bed rest. Persistently low EPO levels with bed rest may participate directly or indirectly to hemolysis in the bone marrow. Erythroid progenitors and early erythroblasts are continuously generated in large excesses and the fraction surviving to complete differentiation is regulated by EPO levels^49^. Haptoglobin binds to free plasma hemoglobin; the unchanged haptoglobin levels in this study supported a predominantly extravascular mechanism of hemolysis^50–52^. Importantly, while low-grade hemolysis physiologically attenuated hemoconcentration, it was an inappropriate response to a low tHb mass (Fig. 3c), which can contribute to anemia.

Bed rest unloads the skeleton and, therefore, the hemopoietic bone marrow. Mechanosensitive osteolineage cells directly interact with hemopoietic stem cells around bone marrow sinusoids^53–55^. While exercise stimulates erythropoiesis, the elimination of axial loading imposed by bed rest may inhibit RBC production^56,57^. Skeletal offloading can also impair hematopoiesis via the bone marrow adipose tissue (MAT)^54,55^. Inactivity increases MAT, which can impair hematopoiesis by occupying space and/or through paracrine action^58^. Tight reciprocal relationship between MAT and hematopoiesis^59,60^ support that adipocytes are negative regulators of hematopoietic stem cell^55^. Low EPO has also been shown to favor adipogenesis^61^. Each marrow adipocyte in the mouse displaced ~30 hematopoietic cells^59^ and could interact with more than 100 through direct contact or via reticular macrophages of the erythroblast islands^62^. Increased MAT can impede the migration of maturing erythroid cells from the erythroblast islands to the sinusoids for egress into circulation^63^ and increase hemolysis locally in the bone marrow. Fluid shift to the bone marrow may, similar to MAT increase ineffective erythropoiesis.

Enforced bed rest disrupts the activity cycle between daytime and nighttime. Aborting circadian oscillations may alter erythrocyte control since both heme synthesis and degradation molecules are central actors in multiple internal clocks and metabolic pathways^64,65^. Heme binding to CLOCK protein regulates circadian control^66^. Both heme biosynthesis enzyme delta-aminolevulinate synthase 1 and hemolysis enzymes HO in liver followed a circadian expression^67–69^. Expression of HO mRNA in the retina peaked in the morning and middle of the night^11^. Klemz showed that rhythmic heme degradation was required for normal circadian rhythms, possibly through CO signaling^70^. Bed rest abolished the oscillatory verticalization for 60 days and may have shifted the circadian cycle-dependent erythropoiesis-hemolysis balance towards increased hemolysis.

At reambulation, hemolysis levels readily returned to baseline; and reversed fluid shift resulted in decreased RBC concentration (Figs 2 and 3). This was accompanied by a strong erythropoietic response with increased EPO levels and reticulocyte concentrations to palliate the low RBC concentration and tHb mass. Both iron and transferrin saturation dropped below normal levels possibly reflecting iron mobilization for intense erythropoiesis and iron losses from blood draws.

Since bed rest HDT is a microgravity analog, persistently increased hemolysis supports continued astronaut monitoring throughout space missions since space anemia worsens with increasing duration of exposure to microgravity^6^.

Beyond applications to space medicine, these results may apply on Earth to patients with reduced mobility^24^ and the elderly^27,28^. These populations spend increased time in bed or in wheelchairs and suffer high prevalence of chronic anemia of unexplained etiology^25–29,71–74^. Our findings of low-grade hemolysis in healthy volunteers with enforced reduced mobility is novel but the study did not include a horizontal bed rest group. However, Mitlyng et al. had identified a similar 25% shorter RBC lifespan in patients with arthritis limiting mobility and with chronic anemia^75^. Therefore, low-grade hemolysis may be the mechanism explaining the high prevalence of anemia in patients who remain bedridden for prolonged durations^76–80^. In these patients, different approaches may be needed to address increased hemolysis with bed rest. Should MAT accumulation be a major contributor, antagonists of the peroxisome proliferator-activated receptor-γ may be favored^81^. Should skeleton unloading or circadian dysregulation be culprits, minimizing time in bed with early mobilization and promoting physical activity may be beneficial. Finally, serum adenosine levels were found to be enhanced with hemolysis and in a hypoxic bedridden environment, and adenosine can modulate important metabolic processes as well as the immune response^82^.

There are several limitations associated with the presented work. Parallel investigations during this bed rest study required venipunctures of 483 ml between baseline and day 60 of bed rest (Supplementary Fig. 3). Despite iron losses arising from the venipunctures, increased iron/transferrin saturation during bed rest indicated its enhanced release. Similarly, venipunctures participated in lowering the tHb mass, which could have lowered CO elimination proportionately. Again, a 23% increase in CO elimination was measured from a 10% smaller pool of RBCs further supporting enhanced hemolysis during bed rest. Concomitant investigations required biopsies of muscle and fat at baseline and bed rest Day 56. Myoglobin and cytochrome degradation may have increased CO elimination. CO at baseline appeared unaffected but biopsies may have contributed to the Day 57 CO elimination data (Fig. 2 and Supplementary Fig. 4). Owing to the small sample size and targeted physiological outcomes, we did not correct for multiple hypothesis-testing. Since all of baseline measures but one were carried out between BDC-4 and BDC-1, they corresponded to the status within 4 days of the bed rest phase.

The ground-based spaceflight analog head-down tilt bed rest triggered enhanced hemolysis measured with all three by-products of heme degradation. Persistent hemolysis over 60 days contributed to lower tHb mass. These results suggest that enhanced hemolysis may constitute one important mechanism linking anemia with microgravity in astronauts and anemia with bed rest on Earth.

## Methods

### Subjects and setting

The study was sponsored by the Center National d’Etudes Spatiales and collaborator the European Space Agency. The study was approved by the Comité de protection des personnes Sud-ouest et outre-mer (ID-RCB:2016-A00401-50), the Ottawa Health Science Network REB (20160925-01H) and registered at ClinicalTrials.gov: NCT03594799 (Date submitted: November 24 2017; Date posted: July 20 2018). Informed consent was obtained from all individual participants included in the study and all participants signed informed consent regarding publication of their data. The study ran at the MEDES space clinic in France between January 2017 and January 2018. A volunteer sample of 20 men were recruited via the clinic website and the media. Inclusion criteria included age (20 to 45years) and BMI (22 to 27 kg/m^2^). Exclusions included hematological diseases, recent blood donation, smokers and active medical treatment (Supplementary Detail 1). The sample size was selected to detect a change in fasting plasma triglycerides concentration with the nutritional intervention. Sixteen research groups carried out various investigations in parallel, which constrained the sampling schedule and explained the variable sampling times between outcome measures.

### Design, exposure, and intervention

This prospective randomized clinical trial tested a nutritional intervention to prevent deconditioning from exposure to bed rest head-down tilt (HDT). The design included 2 identical campaigns of 10 volunteers starting 8 months apart. Each campaign was comprised of 14 days of baseline data collection (BDC), followed by 60 days of bed rest HDT, 14 days of recovery or reambulation (R), with follow-up at R30. The nutritional intervention consisted of half the participants receiving daily 741 mg polyphenols, 2.1 g Omega-3, 168 mg vitamin E and 80 µg selenium (detailed nutritional intervention in Supplementary Detail 2).

### Quantification of hemolysis

We quantified the three by-products of hemoglobin degradation by heme oxygenase. Alveolar and ambient air samples to measure CO concentrations ([CO]) were collected at eight time points, one during baseline data collection (BDC-1), four during bed rest (HDT5, HDT11, HDT30, HDT57), and three during reambulation (R5, R12, R30) (Supplementary Fig. 5). A ninth measure at BDC-14 was dropped due to equipment failure at study outset (Supplementary Fig. 6). CO elimination was performed according to Shahin^83^, which involves replication of the measures, and implemented major improvements over prior methods^15^. Briefly, alveolar and ambient air samples were collected upon waking at 07:00 hours. Alveolar air collection followed 20 s of breath holding. On expiration, the initial 400 ml from large airways was discarded using a pressure-sensitive one-way valve, the remainder flowed to a 750 ml collection bag. Ambient samples from the participants’ rooms were collected simultaneously into separate 750 ml bags. Air samples were analyzed using a gas chromatograph with a reduction gas detector at a precision of 5 ppb (PeakPerformer1RCP, Peak Laboratories, CA)^84^.

Each molecule of biliverdin from heme degradation is conjugated in the liver to bilirubin, excreted in the intestine, converted to urobilinoids and eliminated in the feces^41^. We collected stools over 3-day periods before bed rest (BDC-13,-12,-11), four times during bed rest (HDT11,12,13, HDT30,31,32, HDT56,57,58) and at reambulation (R5,6,7). Stools were protected from light and immediately frozen at −20 °C. Later, each sample was thawed, weighed, homogenized and a 350 mg aliquot was suspended with 16 mM NaCl. Urobilinoids were extracted from 0.4 ml of the suspension according to Kotal^41^. Briefly, the suspension was oxidized by Lugol iodine. Adding 54 mM zinc acetate in dimethyl sulfoxide formed a zinc complex and 82 mM of cysteine was added. The extraction process was repeated twice. The absorption of the zinc complex of oxidized urobilinoids at 508 nm was compared to a standard curve using photometry (Infinite200PRO, Tecan, Switzerland).

Total hemoglobin mass was measured using CO-rebreathing at baseline (BDC-4), at the end of bed rest (HDT60) and at reambulation (R7) according to Burge^85^. Briefly, participants breathed pure oxygen for 5 min before a baseline measure of carboxyhemoglobin. The participants then received an individualized dose of CO (50–90 ml) rebreathed for 10 min before a second measure of carboxyhemoglobin. THb was calculated using carboxyhemoglobin kinetics. Importantly, since exogenous CO washout requires 2–6 h^86–89^, we measured CO elimination at least 3 days after each tHb measures (Supplementary Fig. 4).

Blood samples were collected at 07 h30 min at BDC-4, HDT20, HDT49, R13, and R30 and processed at Laboratoire Biopole, France (Accreditation 31.3.31158.1) for total bilirubin, serum iron, saturation of transferrin, EPO, and haptoglobin. RBC, Hb, and reticulocyte concentrations were sampled at the same time points plus at HDT60 and R1 (Supplementary Fig. 5). Since all baseline measures but one were carried between BDC-4 and BDC-1, they corresponded to the status within 4 days of the bed rest phase. The one measure outside that range was the 3-day stool collection of urobilinoids (from BDC-13 to BDC-11). All measures were performed blindly except for CO where the type of sample (ambient or alveolar) was disclosed in order to calibrate accordingly.

### Statistical methods

There were 16 investigator groups each with primary and secondary outcomes. For our experiment, CO elimination constituted the primary outcome. The effect of nutritional intervention was assessed using Mann Whitney *U*-tests. For the primary outcome measure (CO elimination) and all outcomes, data with 95% confidence interval are presented in Tables 1 and 2. For the primary outcome measure (CO elimination) and all outcomes, change from baseline with 95% confidence interval are presented in Results, and Figs 2 and 3. Paired *t*-test were ran between baseline and experimental time points with no correction for multiple testing.

### Reporting summary

Further information on research design is available in the Nature Research Reporting Summary linked to this article.

## Supplementary information

Supplementary data

Reporting Summary Checklist

## Data Availability

Data will be made available upon reasonable request to the corresponding author.
