# Supplementary material for: Six degrees head-down tilt bed rest caused low-grade hemolysis: a prospective randomized clinical trial
Source: NPJ Microgravity. 2021 Feb 15;7:4. doi: 10.1038/s41526-021-00132-0 (PMC7884785; doi:10.1038/s41526-021-00132-0)
Supplement: Supplementary file 1 — Supplementary data [file 41526_2021_132_MOESM1_ESM.pdf]

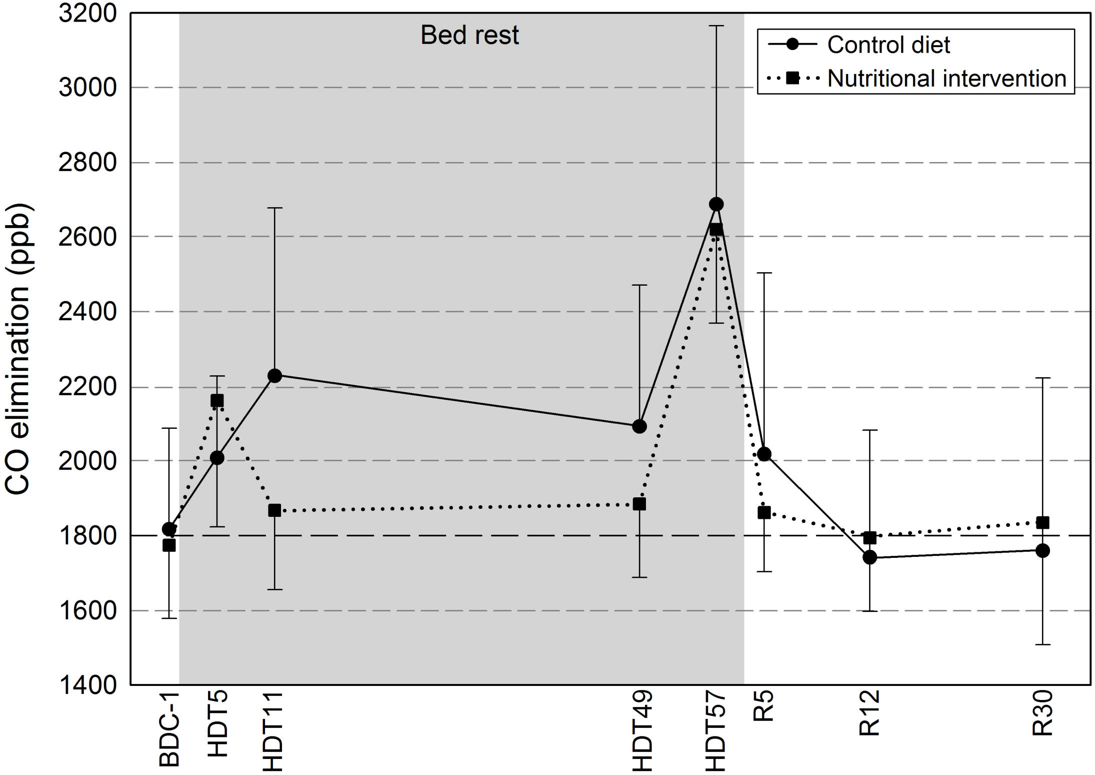

Supplementary Figure 1: Effect of nutritional intervention on CO elimination. The nutritional intervention did not influence endogenous CO elimination, the primary outcome measure of this trial. Shaded area represents the bed rest HDT phase. BDC: Baseline Data Collection; HDT: Head-Down Tilt; R: Recovery or Reambulation. Error bars: 95% Confidence Interval.

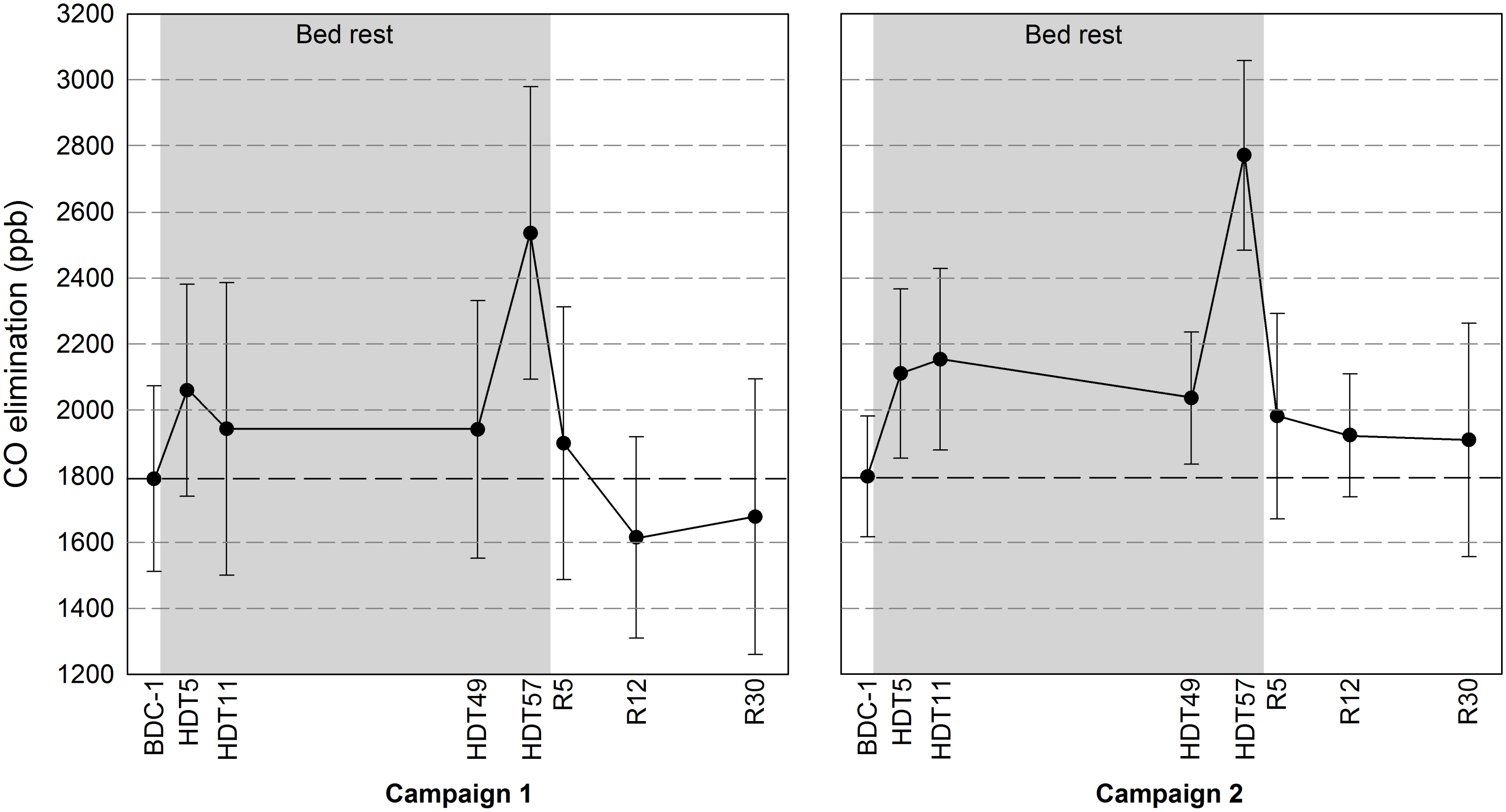

Supplementary Figure 2: Effect of campaign on CO elimination. Both campaigns of 10 volunteers each showed reproducible patterns of endogenous CO elimination, the primary outcome measure of this trial. Shaded area represents the bed rest HDT phase. BDC: Baseline Data Collection; HDT: Head-Down Tilt; R: Recovery or Reambulation. Error bars: 95% Confidence Interval.

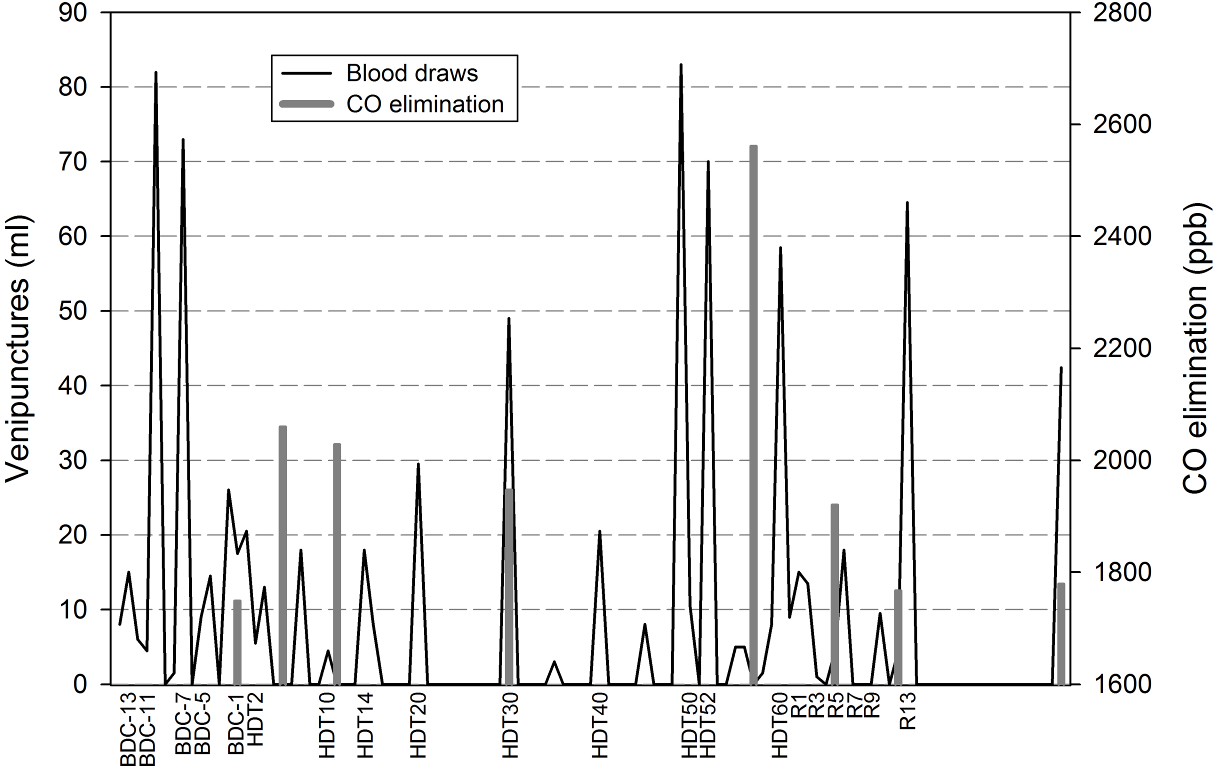

Supplementary Figure 3: Blood was drawn for various experimental investigations before, during and after bed rest. Specifically, between the BDC-4 and HDT60 measures of tHb mass, 483ml of blood were drawn. There was no pattern of association between blood draws and endogenous CO elimination, the primary outcome measure of this trial.

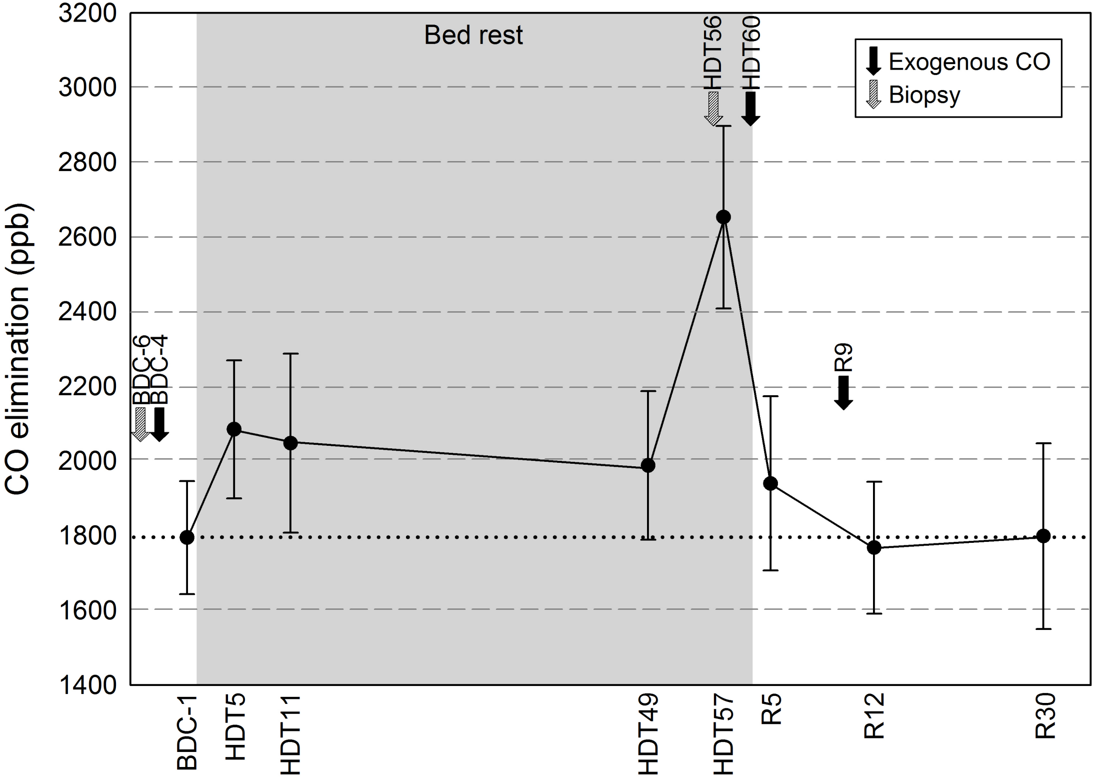

Supplementary Figure 4: Effect of exogenous CO administration and of biopsies on CO elimination. CO was exogenously administered for the measurement of tHb mass thrice during each campaign. Based on exogenous CO elimination kinetics, we measured endogenous CO elimination at least 3 days after each tHb mass measure. Muscle and abdominal fat biopsies occurred twice during each campaign, respectively 5 days and 1 day before CO elimination measures. While the BDC-6 biopsy did not appear to alter CO elimination, the CO elimination at HDT57 may have been influenced by local hematoma and/or the degradation of myoglobin and cytochrome of muscular origin. Shaded area represents the bed rest HDT phase. BDC: Baseline Data Collection; HDT: Head-Down Tilt; R: Recovery or Reambulation. Error bars: 95% Confidence Interval. *$p<0.05$ compared to baseline.

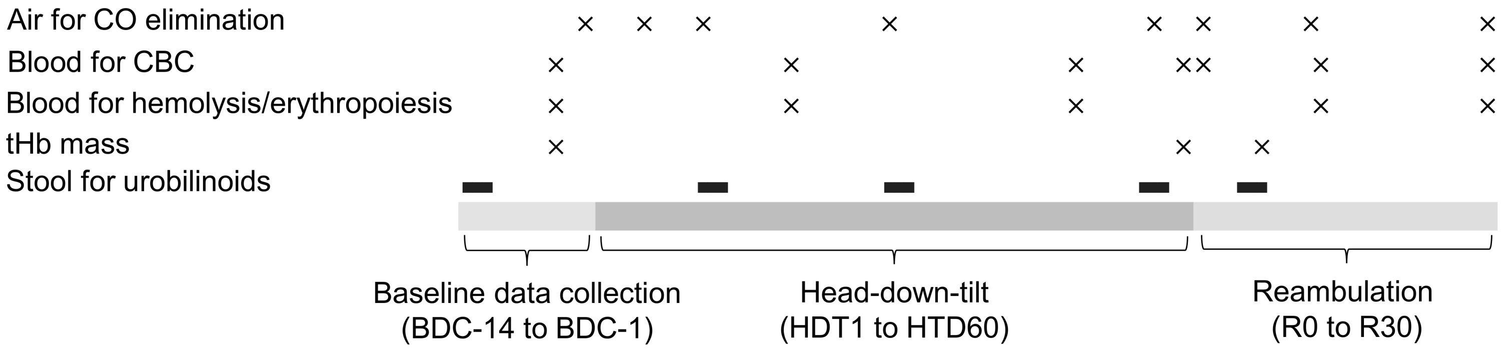

Supplementary Figure 5: Flow chart outlining the chronological phases of the bed rest study as well as sampling schedule for the various outcomes measured. CO: Measure of carbon monoxide elimination; CBC: complete blood count. Hemolysis/erythropoiesis markers included: total bilirubin, iron, transferrin saturation, haptoglobin and EPO. CO elimination was measured at BDC-1, HDT5, HDT11, HDT30, HDT57, R5, R12 and R30. Blood for CBC was collected at BDC-4, HDT20, HDT49, HDT60, R1, R13 and R30. Blood for hemolysis/erythropoiesis markers was collected at BDC-4, HDT20, HDT49, R13 and R30. THb mass was measured at BDC-4, HDT60 and R7. Finally 3-day stool collections for urobilinoids elimination occurred at BDC-13,-12,-11, HDT11,12,13, HDT30,31,32, HDT56,57,58 and R5,6,7.

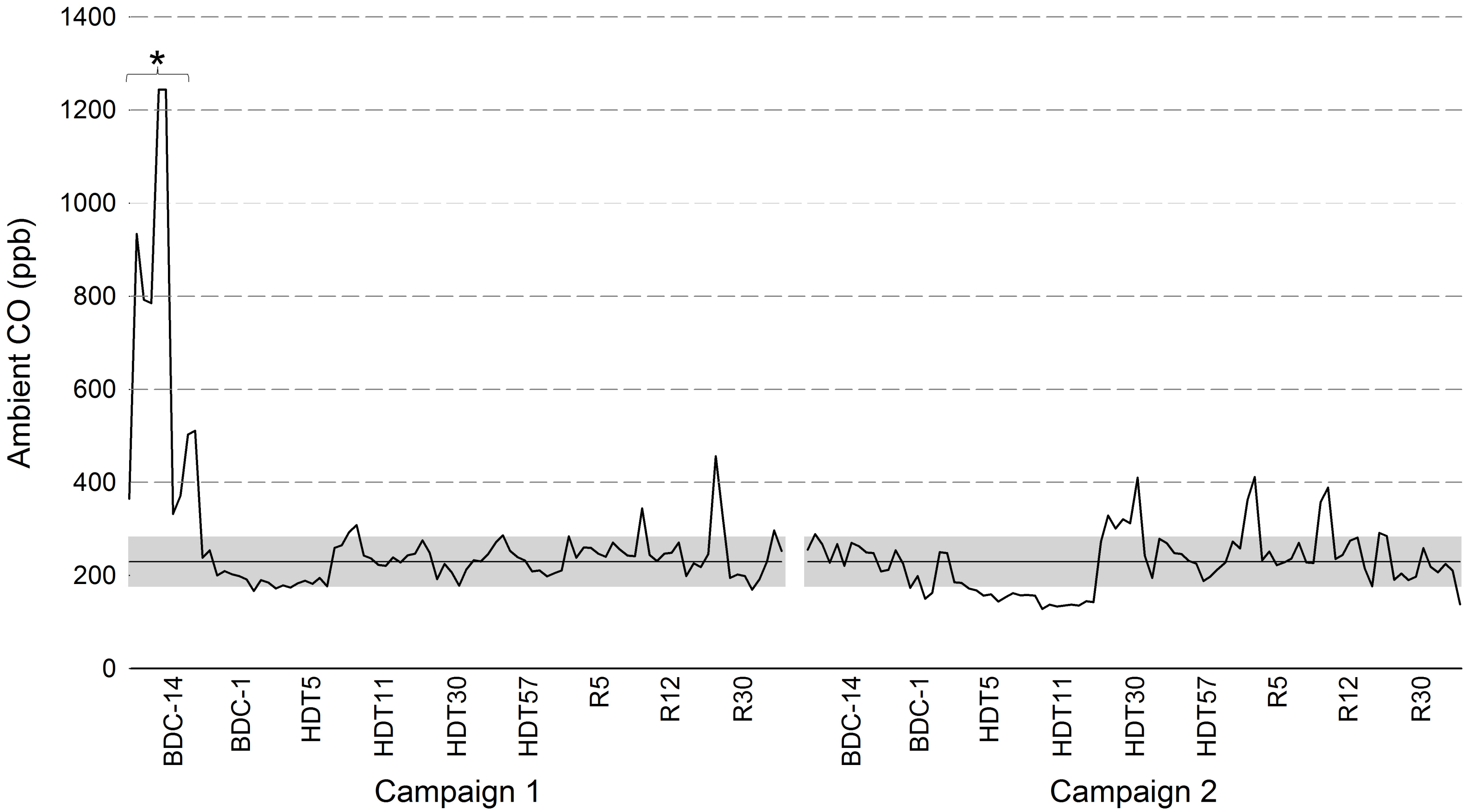

Supplementary Figure 6: CO elimination at BDC-14. A ninth measure of CO elimination was planned in the original protocol but dropped due to equipment failure for the measure of ambient CO. The syringe provided in Europe differed from the North American-made and likely outgased CO leading to large concentrations of ambient air CO at the outset of the study. We modified the methods to collect ambient air directly using the sample bag for all following data collection points. Thereafter, ambient [CO] remained stable throughout the trial. *indicates erroneous ambient [CO] readings, solid line indicates mean ambient air [CO] for both campaigns, shading indicates 1 standard deviation.

Supplementary Table 1: Outcomes showing the effect of nutritional intervention. Data (95% CI) before, during and after bed rest. Shaded columns show the bed rest phase.

Measure		Bedrest study time point																
		BDC-13	BDC-4	BDC-1	HDT5	HDT11	HDT20	HDT30	HDT49	HDT56	HDT57	HDT60	R1	R5	R7	R12	R13	R30
CO ppb	Control			1817 (1545-2088)	2008 (1788-2227)	2228 (1778-2678)			2092 (1713-2472)		2687 (2208-3166)		2018 (1532-2505)			1742 (1403-2082)		1760 (1297-2222)
	Intervention			1774 (1578-1970)	2162 (1824-2500)	1868 (1656-2080)			1885 (1688-2082)		2620 (2369-2871)		1862 (1704-2021)			1794 (1598-1991)		1835 (1508-2162)
Bilirubin mg/l	Control		7.3 (4.6-10)				8.0 (4.8-11.2)		8.0 (4.5-11.5)								7.0 (4.6-9.4)	4.0 (2.8-5.2)
	Intervention		6.1 (4.9-7.3)				7.0 (6.4-7.6)		7.5 (7.0-8.0)								6.1 (5.4-6.8)	6.0 (4.4-7.6)
Urobilinoid mg/24hr	Control	314 (198-430)				445 (258-631)		361 (229-493)		391 (238-544)				349 (210-488)				
	Intervention	339 (248-429)				378 (254-502)		358 (263-454)		360 (198-522)				321 (174-467)				
Iron ug/dl	Control		83.4 (71.6-95.1)				93.0 (72.5-113.5)		85.7 (75.2-96.3)								65.9 (50.8-81.1)	55.1 (46.8-63.5)
	Intervention		79.4 (63.1-95.7)				90.7 (73.3-108.1)		94.7 (77.2-112.2)								69.9 (53.6-86.2)	64.2 (41.2-87.3)
Iron saturation %	Control		30.1 (24.2-36.1)				31.9 (24.1-39.6)		31.3 (27.5-35.2)								24.9 (19.4-30.4)	19.3 (16.3-22.3)
	Intervention		27.8 (20.5-35.1)				31.0 (23.3-38.7)		34.9 (26-43.8)								25.4 (18.6-32.2)	20.6 (12.8-28.4)
RBC 10^6/mm^3	Control		4.479 (4.217-4.741)				4.929 (4.751-5.107)		4.977 (4.675-5.279)			4.797 (4.513-5.081)	4.462 (4.198-4.726)				4.311 (4.014-4.608)	4204286 (3859774-4548798)
	Intervention		4.579 (4.439-4.719)				5.092 (4.903-5.281)		5.036 (4.801-5.270)			4.809 (4.542-5.076)	4.475 (4.284-4.665)				4.385 (4.160-4.610)	4.465 (4.218-4.712)
Reticulocyte 10^3/µl	Control		46.52 (34.86-58.19)				52.50 (38.48-66.52)		47.37 (36.99-57.75)								63.76 (52.90-74.62)	52.28 (46.28-58.27)
	Intervention		48.19 (34.18-62.20)				48.94 (33.13-64.74)		44.45 (33.00-55.90)								64.13 (50.05-78.20)	59.58 (45.69-73.46)
tHb g/kg	Control		10.63 (9.99-11.26)									9.89 (8.90-10.88)			9.51 (8.90-10.13)			
	Intervention		11.20 (10.28-12.12)									10.13 (9.47-10.80)			9.68 (8.80-10.56)			
tHb g	Control		822.99 (724.42-921.56)									748.49 (621.23-875.74)			740.64 (643.93-837.34)			
	Intervention		813.88 (738.23-889.53)									722.93 (655.98-789.89)			708.33 (649.43-767.23)			
EPO mUI/ml	Control		12.4 (9.9-14.8)				8.4 (7.0-9.7)		9.1 (7.4-10.9)								15.0 (10.7-19.3)	16.8 (10.7-23.0)
	Intervention		12.1 (8.5-15.7)				9.4 (7.2-11.7)		11.2 (8.6-13.7)								17.7 (11.3-24.1)	18.5 (11.9-25.2)
Haptoglobin g/l	Control		1.06 (0.89-1.24)				1.04 (0.87-1.20)		1.03 (0.84-1.22)								1.13 (0.95-1.31)	1.14 (0.88-1.40)
	Intervention		0.99 (0.76-1.23)				0.95 (0.73-1.17)		1.04 (0.76-1.32)								1.02 (0.79-1.24)	1.08 (0.8-1.35)
HBG g/100ml	Control		13.5 (13.0-13.9)				15.1 (14.6-15.7)		15.2 (14.4-16.0)			14.6 (13.8-15.3)	13.6 (12.8-14.4)				13.2 (12.4-14.0)	13.0 (12.1-13.8)
	Intervention		13.9 (13.5-14.3)				15.1 (14.7-15.4)		14.8 (14.3-15.3)			14.2 (13.7-14.8)	13.2 (12.8-13.6)				13 (12.5-13.5)	13.3 (12.8-13.9)

Supplementary Detail 1: Detailed list of exclusion criteria

- Past record of orthostatic intolerance,
- Cardiac rhythm disorders,
- Chronic back pains,
- History of hiatus hernia or gastro-esophageal reflux,
- History of thyroid dysfunction, renal stones, diabetes, migraines,
- Past records of thrombophlebitis, family history of thrombosis or positive response in thrombosis screening procedure,
- Allergy including xylocaine allergy
- Abnormal result for lower limbs echo-doppler,
- History or active claustrophobia,
- History of genetic muscle and bone diseases of any kind,
- History of sleep disorders, no shift work or travel across more than one time zone in previous two months
- Vestibular disorders,
- Audition problem,
- Vision corrected no more than 20/30, color-blindness
- Bone mineral density: T-score ≤ -1.5,
- Osteosynthesis material, presence of metallic implants,
- History of knee problems or joint surgery/broken leg
- Poor tolerance to blood sampling,
- Having given blood (more than 8ml/kg) in a period of 8 weeks or less before the start of the experiment,
- Special food diet, vegetarian or vegan, food allergy especially allergic to peanut or soya.
- Positive reaction to any of the following tests: HVA IgM (hepatitis A), HBs antigen (hepatitis B), anti-HVC antibodies (hepatitis C), anti-HIV1+2 antibodies,
- Subject already participating or in the exclusion period of a clinical research,
- Refusal to give permission to contact his general practitioner,
- Incarcerated persons,
- Subject who, in the judgment of the investigator, is likely to be non-compliant during the study,
- or unable to cooperate because of a language problem or poor mental development,
- Subject who has received more than 4500 Euros within 12 months for being a research
- subject
- Subject under guardianship or trusteeship.

Supplementary Detail 2: Nutritional intervention

Natural antioxidant dietary supplement. Half of the participants received a supplement that consisted of a daily dose of approximately 741 mg of bioactive polyphenols (Laboratoires Spiral, Dijon) in 6 pills per day: 2 at breakfast, 2 at lunch and 2 at dinner; approximately 1g of omega-3 acid ethyl esters (Omacor®) in 3 capsules per day: 1 at breakfast, 1 at lunch and 1 at dinner; and 168 mg of vitamin E associated with 80 µg of selenium (Solgar®) in a single pill per day at breakfast.
