# Supplementary material for: Six degrees head-down tilt bed rest caused low-grade hemolysis: a prospective randomized clinical trial
Source: NPJ Microgravity. 2021 Feb 15;7:4. doi: 10.1038/s41526-021-00132-0 (PMC7884785; doi:10.1038/s41526-021-00132-0)
Supplement: Supplementary file 2 — Reporting Summary Checklist [file 41526_2021_132_MOESM2_ESM.pdf]

nature research

Corresponding author(s): DBPR NPJMGRAV-00555

Last updated by author(s): Dec 1, 2020

Reporting Summary

Nature Research wishes to improve the reproducibility of the work that we publish. This form provides structure for consistency and transparency in reporting. For further information on Nature Research policies, see our Editorial Policies and the Editorial Policy Checklist.

Statistics

For all statistical analyses, confirm that the following items are present in the figure legend, table legend, main text, or Methods section.

n/a	Confirmed	
☐	☒	The exact sample size (n) for each experimental group/condition, given as a discrete number and unit of measurement
☐	☒	A statement on whether measurements were taken from distinct samples or whether the same sample was measured repeatedly
☐	☒	The statistical test(s) used AND whether they are one- or two-sided<br>*Only common tests should be described solely by name; describe more complex techniques in the Methods section.*
☒	☐	A description of all covariates tested
☐	☒	A description of any assumptions or corrections, such as tests of normality and adjustment for multiple comparisons
☐	☒	A full description of the statistical parameters including central tendency (e.g. means) or other basic estimates (e.g. regression coefficient) AND variation (e.g. standard deviation) or associated estimates of uncertainty (e.g. confidence intervals)
☒	☐	For null hypothesis testing, the test statistic (e.g. F, t, r) with confidence intervals, effect sizes, degrees of freedom and P value noted<br>*Give P values as exact values whenever suitable.*
☒	☐	For Bayesian analysis, information on the choice of priors and Markov chain Monte Carlo settings
☒	☐	For hierarchical and complex designs, identification of the appropriate level for tests and full reporting of outcomes
☒	☐	Estimates of effect sizes (e.g. Cohen's d, Pearson's r), indicating how they were calculated

Our web collection on statistics for biologists contains articles on many of the points above.

Software and code

Policy information about availability of computer code

Data collection	No software was used
Data analysis	No software was used

For manuscripts utilizing custom algorithms or software that are central to the research but not yet described in published literature, software must be made available to editors and reviewers. We strongly encourage code deposition in a community repository (e.g. GitHub). See the Nature Research guidelines for submitting code & software for further information.

Data

Policy information about availability of data

All manuscripts must include a data availability statement. This statement should provide the following information, where applicable:

- Accession codes, unique identifiers, or web links for publicly available datasets
- A list of figures that have associated raw data
- A description of any restrictions on data availability

Data will be made available upon reasonable request to the corresponding author.

Field-specific reporting

Please select the one below that is the best fit for your research. If you are not sure, read the appropriate sections before making your selection.

☒ Life sciences ☐ Behavioural & social sciences ☐ Ecological, evolutionary & environmental sciences

For a reference copy of the document with all sections, see nature.com/documents/nr-reporting-summary-flat.pdf

Life sciences study design

All studies must disclose on these points even when the disclosure is negative.

Sample size	The sample size was selected by the study sponsor (Centre National d'Etudes Spatiales) to detect a change in fasting plasma triglycerides concentration with the nutritional intervention.
Data exclusions	Exclusions are detailed in the CONSORT flow diagram. 64 participants were assessed for eligibility of which 44 were excluded for not meeting the inclusion criteria (detailed in the supplementary material). CO data from 1 participant at R30 was excluded due to admitted environmental factors (cigarette smoking). Blood was not drawn on three participants at reambulation Day 30. Stool samples were not collected for 1 participant at HDT11,12,13. Total Hb mass determination was excluded for 3 participants due to technical difficulties.
Replication	Methods for the primary outcome measure (CO elimination) were replicated and secondary outcome measures were clinical laboratory results or standard protocols.
Randomization	Participants were randomized to the nutritional intervention or control group.
Blinding	All measures were preformed blindly except for CO where the type of sample (ambient or alveolar) was disclosed in order to calibrate accordingly.

Reporting for specific materials, systems and methods

We require information from authors about some types of materials, experimental systems and methods used in many studies. Here, indicate whether each material, system or method listed is relevant to your study. If you are not sure if a list item applies to your research, read the appropriate section before selecting a response.

Materials & experimental systems

n/a	Involved in the study	
☒	☐	Antibodies
☒	☐	Eukaryotic cell lines
☒	☐	Palaeontology and archaeology
☒	☐	Animals and other organisms
☐	☒	Human research participants
☐	☒	Clinical data
☒	☐	Dual use research of concern

Methods

n/a	Involved in the study	
☒	☐	ChIP-seq
☒	☐	Flow cytometry
☒	☐	MRI-based neuroimaging

Human research participants

Policy information about studies involving human research participants

Population characteristics	A volunteer sample of 20 men were recruited for the study. Inclusion criteria included age (20 to 45years) and BMI (22 to 27kg/m2). Exclusions included hematological diseases, recent blood donation, smokers and active medical treatment (Supplementary Detail 1)
Recruitment	Clinic website and the media.
Ethics oversight	The study was approved by the Comité de protection des personnes Sud-ouest et outre-mer (ID-RCB:2016-A00401-50), the Ottawa Health Science Network REB (20160925-01H).

Note that full information on the approval of the study protocol must also be provided in the manuscript.

Clinical data

Policy information about clinical studies

All manuscripts should comply with the ICMJE guidelines for publication of clinical research and a completed CONSORT checklist must be included with all submissions.

Clinical trial registration	Registered at ClinicalTrials.gov: NCT03594799 (Date submitted: November 24 2017; Date posted: July 20 2018).
Study protocol	Details of the study protocol are also available at ClinicalTrials.gov: NCT03594799
Data collection	The study ran at the MEDES space clinic in France between January 2017 and January 2018.
Outcomes	The primary outcome measure was alveolar CO concentration. Secondary outcome measures were total bilirubin, iron, urobilinoids, transferrin saturation, RBC concentration, EPO, total hemoglobin mass, and reticulocyte concentration.
